# Enhancing Public Health Communication Regarding Vaccine Trials: Design and Development of the Pan-European VACCELERATE Toolkit

**DOI:** 10.2196/44491

**Published:** 2023-04-03

**Authors:** Christos D Argyropoulos, Janina Leckler, Jon Salmanton-García, Marinos Constantinou, Alexandra Alexandrou, Sophia Themistocleous, Evgenia Noula, George Shiamakkides, Andria Nearchou, Fiona A Stewart, Kerstin Albus, Markela Koniordou, Ioannis Kopsidas, Orly Spivak, Margot Hellemans, Greet Hendrickx, Ruth Joanna Davis, Anna Maria Azzini, Paula Valle Simon, Antonio Javier Carcas-Sansuan, Helena Hervius Askling, Sirkka Vene, Jana Baranda Prellezo, Elena Álvarez-Barco, Alan J Macken, Romina Di Marzo, Catarina Luís, Ole F Olesen, Jesus A Frias Iniesta, Imre Barta, Krisztina Tóth, Murat Akova, Marc M J Bonten, Miriam Cohen-Kandli, Rebecca Jane Cox, Lenka Součková, Petr Husa, Ligita Jancoriene, Odile Launay, Jens Lundgren, Patrick Mallon, Charis Armeftis, Laura Marques, Pontus Naucler, Jordi Ochando, Evelina Tacconelli, Pierre van Damme, Theoklis Zaoutis, Sanne Hofstraat, Patricia Bruijning-Verhagen, Markus Zeitlinger, Oliver A Cornely, Zoi Dorothea Pana

**Affiliations:** 1 School of Medicine European University Cyprus Nicosia Cyprus; 2 Cologne Excellence Cluster on Cellular Stress Responses in Aging-Associated Diseases Faculty of Medicine and University Hospital Cologne University of Cologne Cologne Germany; 3 Center for Integrated Oncology Aachen Bonn Cologne Duesseldorf and Excellence Center for Medical Mycology Department I of Internal Medicine, Faculty of Medicine and University Hospital Cologne University of Cologne Cologne Germany; 4 Collaborative Center for Clinical Epidemiology and Outcomes Research Athens Greece; 5 Ministry of Health of Israel Jerusalem Israel; 6 VAXINFECTIO, Centre of Evaluation of Vaccination Faculty of Medicine and Health Science Universiteit Antwerpen Antwerp Belgium; 7 Infectious Diseases, Department of Diagnostic and Public Health University of Verona Verona Italy; 8 Hospital La Paz Institute for Health Research Madrid Spain; 9 Servicio Madrileño de Salud Madrid Spain; 10 Department of Infectious Diseases Karolinska University Hospital Stockholm Sweden; 11 Department of Medicine Division of Infectious Diseases Karolinska Institutet Stockholm Sweden; 12 Centro Nacional de Microbiología Instituto de Salud Carlos III Madrid Spain; 13 Centre for Experimental Pathogen Host Research School of Medicine University College Dublin Dublin Ireland; 14 European Vaccine Initiative Heidelberg Germany; 15 National Koranyi Institute for Pulmonology Budapest Hungary; 16 Department of Infectious Diseases and Clinical Microbiology Faculty of Medicine Haceteppe University Ankara Turkey; 17 Julius Center for Health Sciences and Primary Care University Medical Centre Utrecht, Utrecht University Utrecht Netherlands; 18 Department of Medical Microbiology University Medical Centre Utrecht Utrecht Netherlands; 19 Influenza Centre, Department of Clinical Science University of Bergen Bergen Norway; 20 Masaryk University Brno Czech Republic; 21 University Hospital Brno Brno Czech Republic; 22 Czech Clinical Research Infrastructure Network Brno Czech Republic; 23 Institute of Clinical Medicine Medical Faculty Vilnius University Vilnius Lithuania; 24 Vilnius University Hospital Santaros klinikos Medical Faculty Vilnius University Vilnius Lithuania; 25 Institut National de la Santé et de la Recherche Médicale - France Recherche Nord & Sud Sida- HIV Hépatites Maladies Infectieuses Émergentes Paris France; 26 Assistance Publique Hopitaux de Paris Université Paris Cité Paris France; 27 Centre of Excellence for Health, Immunity and Infections Rigshospitalet, University of Copenhagen Copenhagen Denmark; 28 Centro Hospitalar Universitário do Porto Porto Portugal; 29 University Medical Centre Utrecht Utrecht University Utrecht Netherlands; 30 Medizinische Universität Wien Vienna Austria; 31 Clinical Trials Centre Cologne Faculty of Medicine and University Hospital Cologne University of Cologne Cologne Germany; 32 Center for Molecular Medicine Cologne Faculty of Medicine and University Hospital Cologne University of Cologne Cologne Germany; 33 German Centre for Infection Research Partner Site Bonn-Cologne Cologne Germany

**Keywords:** vaccine trials, volunteer registry, educational material, promotional material, patient education, health communication, health promotion, public health, COVID-19, coronavirus, SARS-CoV-2, pandemic, vaccine, vaccination, hesitancy, campaign, misinformation

## Abstract

**Background:**

The pan-European VACCELERATE network aims to implement the first transnational harmonized and sustainable vaccine trial Volunteer Registry, being a single entry point for potential volunteers of large-scale vaccine trials across Europe. This work exhibits a set of harmonized vaccine trial–related educational and promotional tools for the general public, designed and disseminated by the pan-European VACCELERATE network.

**Objective:**

This study primarily aimed to design and develop a standard toolkit to increase positive attitudes and access to trustworthy information for better access and increased recruitment to vaccine trials for the public. More specifically, the produced tools are focused on inclusiveness and equity, and are targeting different population groups, including underserved ones, as potential volunteers for the VACCELERATE Volunteer Registry (older individuals, migrants, children, and adolescents). The promotional and educational material is aligned with the main objectives of the Volunteer Registry to increase public literacy and awareness regarding vaccine-related clinical research or trials and trial participation, including informed consent and legal issues, side effects, and frequently asked questions regarding vaccine trial design.

**Methods:**

Tools were developed per the aims and principles of the VACCELERATE project, focusing on trial inclusiveness and equity, and are adjusted to local country-wise requirements to improve public health communication. The produced tools are selected based on the cognitive theory, inclusiveness, and equity of differently aged and underrepresented groups, and standardized material from several official trustworthy sources (eg, COVID-19 Vaccines Global Access; the European Centre for Disease Prevention and Control; the European Patients’ Academy on Therapeutic Innovation; Gavi, the Vaccine Alliance; and the World Health Organization). A team of multidisciplinary specialists (infectious diseases, vaccine research, medicine, and education) edited and reviewed the subtitles and scripts of the educational videos, extended brochures, interactive cards, and puzzles. Graphic designers selected the color palette, audio settings, and dubbing for the video story-tales and implemented QR codes.

**Results:**

This study presents the first set of harmonized promotional and educational materials and tools (ie, educational cards, educational and promotional videos, extended brochures, flyers, posters, and puzzles) for vaccine clinical research (eg, COVID-19 vaccines). These tools inform the public about possible benefits and disadvantages of trial participation and build confidence among participants about the safety and efficacy of COVID-19 vaccines and the health care system. This material has been translated into several languages and is intended to be freely and easily accessible to facilitate dissemination among VACCELERATE network participant countries and the European and global scientific, industrial, and public community.

**Conclusions:**

The produced material could help fill knowledge gaps of health care personnel, providing the appropriate future patient education for vaccine trials, and tackling vaccine hesitancy and parents’ concerns for potential participation of children in vaccine trials.

## Introduction

VACCELERATE [1] is an independent, innovative, and transparent Pan-European academic network with the aim of harmonizing multinational vaccine trial initiatives and conducting capacity mapping of vaccine clinical trials sites and laboratories with standardized methods and protocols across Europe. The network identifies and provides access to state-of-the-art vaccine trial sites to accelerate the development of vaccines and recruit volunteers for vaccine trials through the VACCELERATE Volunteer Registry [2].

The main goal of the VACCELERATE Volunteer Registry [2] is to establish the first transnational harmonized trial participation platform serving as single entry point for the European region. The VACCELERATE Volunteer Registry [2], under the mandate of the European Union’s Health Emergency Preparedness and Response Authority (HERA) [3], provides a sustainable platform source for the recruitment of potential volunteers for clinical studies for the current COVID-19 pandemic and future epidemics.

According to the literature, one of the main challenges of a volunteer registry is the registration of a large number of potential trial participants with great diversity [4,5] and dedication in order to be able to recruit and match suitable candidates for the selected clinical trial. Therefore, a great amount of effort is spent looking for strategies to improve communication [6] and convince [7] potential volunteers to take part in vaccine trials.

The enrollment of volunteers in vaccine trials is also influenced by factors such as complacency, conspiracy theories [8] on social media regarding COVID-19, convenience and confidence (the “Three Cs” model of vaccine hesitancy) [7,9], disinformation [10], fake news [11], fear [12], and increasing level of health misinformation [13-15]. The latter leads to an infodemic [16] of misleading information with immediate effects on public health, refusal, and vaccine hesitancy [9] for participation in clinical trials [17,18]. It should be noted that health misinformation is responsible for increased fear and lack of trust, which are major factors for enrollment also in cancer clinical trials [19-21]. Recently, Luís et al [22] reported that there is a lack of public information about COVID-19 vaccine trials among several population groups (eg, minorities and underrepresented populations or individuals), while the available tools for trial participation were also sparse.

The main purpose of this study is the design and development of a standardized toolkit based on the Social Cognitive Theory [23,24] to increase positive attitudes, access to trustworthy information for better access and increased recruitment to vaccine trials. Our secondary objective is to foster engagement of the community through the VACCELERATE Volunteer Registry and smart technology for participating in forthcoming clinical trials [25-28]. The VACCELERATE toolkit has been translated into several languages and is freely and easily accessible to facilitate dissemination among the participating countries of the VACCELERATE Consortium [1].

## Methods

The development of the VACCELERATE educational and promotional tools included the following steps: (1) conceptualization of the idea and context based on the selected educational aims and objectives; (2) selection of targeted population groups (eg, older individuals, children, adolescents, and minorities); (3) prioritization of trustworthy sources and websites for seeking information about COVID-19 vaccine trials for the developed materials; (4) performing graphical design (eg, visualization, audio, product, print, and animation design); and (5) acquisition of a digital object identifier (DOI) for each produced material from a DOI registration agency. More details on the developed tools are presented in the *Results* section.

The context of the educational and promotional tools was created and reviewed by a group of experts in the field of infectious diseases and vaccine and pedagogical research. Two graphic designers developed and selected the appropriate color palette and audio (music, speaker voice, and dubbing) for the video story-tales and they adapted the produced material into an efficient pedagogical, attractive, and eye-catching way. The developed tools were (1) icons related to the COVID-19 pandemic (eg, use of a face mask, syringe, microscope, vaccine, handwashing, virus, etc); (2) different versions of logotypes for the VACCELERATE project; (3) typefaces; (4) samples of fonts with different sizes; (5) color palettes (primary and secondary colors); (6) imagery templates and guidelines for social media (eg, Twitter and Instagram); (7) human characters (ie, medical staff, minorities, and older individuals) as prototypes for any future VACCELERATE promotional and or educational material. Special attention was also paid to foster inclusiveness among the characters (different age groups, different ethic and other minority groups, citizens with disabilities, migrants, etc). The provided tools also include QR codes with a URL. The selected URL can be a website (as shown on the VACCELERATE website [29]) or selected animation videos from trustworthy sources of information (eg, COVID-19 Vaccines Global Access [ie, COVAX]; the European Centre for Disease Prevention and Control; the European Patients’ Academy on Therapeutic Innovation; the European Vaccine Initiative; Gavi, the Vaccine Alliance; the United Nations International Children's Emergency Fund; and the World Health Organization).

In addition, the produced puzzles test memory in a team-based manner and help develop hand-eye coordination.

Finally, the developed tools have been designed in a such a way that can be adjusted in accordance with local country-wise requirements and requirements to build confidence among participants about the safety and efficacy of COVID-19 vaccines and the health care system, which is a key parameter to improve recruitment among minority communities.

## Results

### VACCELERATE Flyers

The flyers (see Multimedia Appendix 1) provide the appropriate amount of information to the public regarding the registration process and the VACCELERATE Volunteer Registry in general. The flyers are A3-sized (29.7×43.18 cm) and include a short description of the VACCELERATE Volunteer Registry objectives and volunteer selection process. In addition, they contain the appropriate contact details (email ID) and link (along with a QR code) for registration in the Volunteer Registry [30]. The registration link directs the interested volunteer to a brief survey (which takes 5-10 minutes to complete) that collects comprehensive information required for clinical trials. The flyer invites all citizens to register, including those with prior COVID-19 vaccination, with specific questions regarding COVID-19 infection, and the invitations to eligible volunteers for future vaccine trials will be sent out by the Coordination Office of VACCELERATE (University Hospital Cologne, Cologne, Germany). Finally, the flyers have been translated into 9 languages (Cypriot Greek, English, French, German, Greek, Hebrew, Italian, Portuguese, and Spanish).

### VACCELERATE Posters

The developed posters (see Multimedia Appendix 1) are A0-sized (84.1×118.9 cm) and depict human characters (ie, medical staff, minorities, and older individuals, among others) and a QR code including a URL for the VACCELERATE Volunteer Registry website [30], as well as catchy slogans such as “Be the missing piece” and “Together we can tackle the COVID-19 pandemic.” Finally, the posters have been translated into 7 languages, namely English, French, German, Greek, Hebrew, Italian, and Spanish, in collaboration with individual partners and national coordinators of the VACCELERATE Consortium. The promotion of the VACCELERATE Volunteer Registry for participating in COVID-19 vaccine trials could be achieved by affixing these prototype posters to a wall at sites or in buildings with high visibility (eg, hospitals, universities, malls, and public buildings).

### VACCELERATE Extended Brochure (Booklet)

The extended VACCELERATE brochure (see Multimedia Appendix 1) comprises valuable information regarding the VACCELERATE Volunteer Registry, such as “What is the VACCELERATE Network,” the mission and objectives, the current promotion activities, the importance of registration, and a description of the registration process. The brochure also contains key information for the volunteers, such as the possibility to withdraw their registration at any time. Finally, the extended brochure also includes a QR code that directs the interested reader to the VACCELERATE Volunteer Registry website [30] after scanning it with a smart device.

### VACCELERATE Interactive Educational Cards

The educational cards enhance general knowledge and address the misinformation about COVID-19 vaccines, vaccine trials, and COVID-19 vaccination, as well as voluntary trial participation of children and adults. Each set of educational cards represents an educational aim and comprises a specific number of cards and target groups, as shown in Table 1.

The interactive educational cards (Multimedia Appendix 1) have been prepared with the help of 2 graphic designers and under the guidance of a pediatrician and several VACCELERATE participants. The educational cards include a QR code with a URL for promoting educational material on the one side and additional text script and characters on the other. The URL can be a website (eg, the one showed on the VACCELERATE website [29]) or selected videos from trustworthy sources (eg, COVID-19 Vaccines Global Access [ie, COVAX]; the European Centre for Disease Prevention and Control; the European Patients’ Academy on Therapeutic Innovation; the European Vaccine Initiative; Gavi, the Vaccine Alliance; the United Nations International Children's Emergency Fund; and the World Health Organization) and produced animation videos from VACCELERATE.

**Table 1:** Characteristics of interactive educational cards.

Set	Cards, n	Target group	Aim
1	25	Individuals aged >12 years	Increase literacy about vaccine trials, trial registries, participation in vaccine trials, and participant procedures in trials
2	10	Individuals aged ≥12 years	Increase literacy about COVID-19 vaccination and messenger RNA vaccines
3	7	Individuals aged ≥12 years	Increase literacy about vaccine manufacturing, the vaccine approval process, safety monitoring, and awareness against vaccine misinformation
4	4	All individuals irrespective of age	Improve awareness and information about the VACCELERATE Volunteer Registry
5	12	All individuals irrespective of age	Increase literacy about COVID-19 vaccination, expand awareness for COVID-19 vaccine equity, and utilitarianism

### VACCELERATE Puzzles

The VACCELERATE puzzles (Multimedia Appendix 1) were developed using graphic techniques under the guidance of the VACCELERATE participants, including a pediatrician, with focus on younger children (aged <12 years) in order to raise their awareness of clinical trials, vaccines, and participation in trials and to promote inclusiveness. The link to the produced puzzles is provided in Multimedia Appendix 1.

One of the developed puzzles comprises 25 pieces and depicts a variety of human characters (ie, medical staff, minorities, older individuals, and children, among others) obtained from the VACCELERATE toolkit, including the appropriate link to the VACCELERATE Volunteer Registry [30]. The concept herein is to include as many human characters as possible to highlight the importance of inclusiveness and diversity in vaccine trials, especially for underserved population groups such as racial and ethnic minorities and older individuals.

The other 2 smaller puzzles (8 pieces each) were developed to familiarize children with symbols and icons related to the COVID-19 pandemic, as well as to understand and recognize the effectiveness of vaccines in minimizing the risk of infection and harmful effects of COVID-19.

### VACCELERATE Animation Videos

#### Educational Videos (Adult and Pediatric)

The first produced educational video (see Multimedia Appendix 1) was mainly focused on adults and designed using advanced animation techniques, appropriate subtitles based on a storyboard and educational aims, and audio settings (eg, music and a speaker voice). In addition, the video included the following sections: (1) highlight and increase information regarding clinical trials and their necessity and usefulness for public health (eg, good clinical practices, vaccine trial phases and monitoring, and applied safety protocols); (2) the contribution of volunteers (ie, citizens, patient advocacy groups, and underserved populations) to this effort for capacity mapping and building of registries; and (3) volunteer safety, benefits, and any potential risks during a clinical trial.

The second educational video (see Multimedia Appendix 1) was mainly aimed at children and adolescents. Special attention was given to contain informative animations while delivering the learning aim and keeping each video segment short. The produced video included the following sections: (1) provide information about the significance of vaccine trials; (2) share knowledge and information about the high value of vaccination as the most powerful “weapon” to prevent morbidity and mortality associated with infectious diseases; (3) acknowledge the advancements in technology and safety procedures, which have contributed significantly to the development, evaluation, approval, and monitoring of safe and effective vaccines; and (4) highlight the contribution and importance of pediatric volunteers in vaccine clinical trials and ensure their safety and the benefits and risks of participating in clinical trials.

#### Promotional Video

The promotional video (see Multimedia Appendix 1) presents short, recorded videos by the VACCELERATE work package leaders and the National Coordinator’s pictures to promote, inform, and describe the VACCELERATE Volunteer Registry through specific text scripts. The aim of the video is the inclusiveness and representation of all participating European Union countries and European Union–associated countries of the VACCELERATE Consortium. Currently, we have active representation from 17 countries (Austria, Belgium, Cyprus, Czech Republic, Germany, Greece, Ireland, Israel, Italy, Hungary, Lithuania, the Netherlands, Norway, Portugal, Spain, Sweden, and Turkey) in the VACCELERATE Volunteer Registry.

## Discussion

### Principal Findings

This paper presents the first set of harmonized promotional and educational tools of the VACCELERATE Volunteer Registry [2]. The produced material, based on the selected population group, includes flyers, posters, interactive educational cards, puzzles, and animation videos. The tools can be downloaded from the official website of VACCELERATE [29] and appropriate references, based on their own DOIs. It should be noted that the provided educational material can be adopted to the user needs and may also be used as base-prototypes for further improvement and production of related tools.

To ensure high rates of vaccination in populations [31] and a large number of participants in vaccine trials, it is necessary to ensure effective, targeted, and trustworthy educational and promotional tools regarding vaccines and vaccine trials, guaranteeing high diversity and inclusiveness in vaccine trials without underrepresentation of minorities.

The developed standardized toolkit is one example of information from trustful sources that can be used against health misinformation and disinformation, lack of trust, fear, and fake news. Furthermore, it can be also used to increase literacy and understanding of the importance of vaccine clinical trials in the general population as part of an emergency preparedness plan.

In addition, the promotional and educational material will be able to bridge the gap in public information (eg, communication approach, target audiences, and type of media produced) regarding COVID-19 vaccine trials, which is covered in only 2.51% of the collected media and only in 4 languages (English, French, German, and Czech) [22]. Another challenge is using visual material and stories in order to inform children about the procedures, importance of, and volunteer participation in vaccine trials and about vaccination.

### Limitations

The impact and assessment of the promotional and educational material are beyond the scope of this study. Nevertheless, we are currently in the process of evaluating them for vaccine trials during this year by another expert group (WP4) of the VACCELERATE Consortium.

### Conclusions

Communicating with trustworthy information to the public, related to public health issues and vaccine research or trials, constitutes the most important preventive strategy against infectious diseases and threats of future emerging infection diseases.

Future initiatives could involve further improvement of the dissemination process for the developed tools in order to increase the number of volunteer registrations and in particular among population groups, which remain underrepresented in vaccine trials. Another initiative could be the design of an innovative video game [31,32] targeting training and educational material for vaccine clinical trials, addressing vaccine hesitancy and refusal.

Other prospective initiatives could be the conduct of studies based on citizen science methods (eg, community-based participatory action research [28]) for vaccine research [25-27,33] and future epidemics by engaging community members and service providers as partners in the research process and providing them the appropriate educational tools for tackling current and future public health issues. Such actions will eventually contribute to reduce the knowledge gaps and hesitancy regarding participation in vaccine trials in the general population.

## Appendix

Multimedia Appendix 1 Information on the promotional and educational material including DOI number, format, title, and language.

## Abbreviations

DOI: Digital Object Identifier
ECDC: European Centre for Disease Prevention and Control
HERA: Health Emergency Preparedness and Response Authority

## Data Availability

All data generated or analyzed during this study are included in this published article and its supplementary information file (Multimedia Appendix 1).
