# Supplementary material for: Enhancing Public Health Communication Regarding Vaccine Trials: Design and Development of the Pan-European VACCELERATE Toolkit
Source: JMIR Public Health Surveill. 2023 Apr 3;9:e44491. doi: 10.2196/44491 (PMC10131613; doi:10.2196/44491)
Supplement: Multimedia Appendix 1 [file publichealth_v9i1e44491_app1.docx]

# Multimedia appendix1. Information on the promotional and educational material including DOI number, format, title, and language.

| **DOI** | **Format** | **Title** | **Language** |
| --- | --- | --- | --- |
| [10.4126/FRL01-006433022](https://doi.org/10.4126/FRL01-006433022) | Educational Cards | Educational Cards - Set 1 | English |
| [10.4126/FRL01-006433024](https://doi.org/10.4126/FRL01-006433024) | Educational Cards | Educational Cards - Set 2 | English |
| [10.4126/FRL01-006433026](https://doi.org/10.4126/FRL01-006433026) | Educational Cards | Educational Cards - Set 3 | English |
| [10.4126/FRL01-006433028](https://doi.org/10.4126/FRL01-006433028) | Educational Cards | Educational Cards - Set 4 | English |
| [10.4126/FRL01-006433030](https://doi.org/10.4126/FRL01-006433030) | Educational Cards | Educational Cards - Set 5 | English |
| [10.4126/FRL01-006432946](https://doi.org/10.4126/FRL01-006432946) | Extended Brochure | Registr Dobrovolníků: VACCELERATE: staňte se chybějícím dílem skládačky - společně se můžeme vypořádat s pandemií COVID-19 - extended brochure Czech | Czech |
| [10.4126/FRL01-006432947](https://doi.org/10.4126/FRL01-006432947) | Extended Brochure | Registre des volontaires pour les essais du vaccin COVID-19: "VACCELERATE - un point d' entrée unique pour l' Europe": local poster French | French |
| [10.4126/FRL01-006432952](https://doi.org/10.4126/FRL01-006432952) | Extended Brochure | Registro de Voluntarios de: VACCELERATE: sé la pieza que falta - juntos podemos afrontar la pandemia de COVID-19 - extended brochure Spanish | Spanish |
| [10.4126/FRL01-006432948](https://doi.org/10.4126/FRL01-006432948) | Extended Brochure | VACCELERATE Probandenregister: sei das fehlende Puzzleteil - gemeinsam können wir die Corona-Pandemie bekämpfen - extended brochure German | German |
| [10.4126/FRL01-006432951](https://doi.org/10.4126/FRL01-006432951) | Extended Brochure | VACCELERATE Registro dei Volontari: sii l’elemento mancante - insieme possiamo combattere la pandemia da COVID-19 - extended brochure Italian | Italian |
| [10.4126/FRL01-006432945](https://doi.org/10.4126/FRL01-006432945) | Extended Brochure | VACCELERATE Volunteer Registry: be the missing piece - together we can tackle the COVID-19 pandemic - extended brochure English | English |
| [10.4126/FRL01-006432949](https://doi.org/10.4126/FRL01-006432949) | Extended Brochure | Μητρώο Εθελοντών : VACCELERATE: Γίνε και εσύ κοµµάτι αυτής της προσπάθειας - Μαζί µπορούµε να αντιµετωπίσουµε την πανδηµία COVID-19 - extended brochure Greek | Greek |
| [10.4126/FRL01-006432950](https://doi.org/10.4126/FRL01-006432950) | Extended Brochure | מאגר המתנדבים של וקסלרייט : VACCELERATE: כך תוכל/י לעזור להשלים את הפאזל - יחד נביא לסיום מגיפת הקורונה - extended brochure Hebrew | Hebrew |
| [10.4126/FRL01-006433010](https://doi.org/10.4126/FRL01-006433010) | Local Poster | Probandenregister für COVID-19-Impfstoffstudien: "VACCELERATE - eine Anlaufstelle für Europa": local poster German | German |
| [10.4126/FRL01-006433000](https://doi.org/10.4126/FRL01-006433000) | Local Poster | Probandenregister für COVID-19-Impfstoffstudien: „VACCELERATE - eine Anlaufstelle für Europa“: local poster Austria | German |
| [10.4126/FRL01-006433018](https://doi.org/10.4126/FRL01-006433018) | Local Poster | Registo de Voluntários para Ensaios Clínicos de Vacinação contra o COVID-19: "VACCELERATE - um único ponto de entrada para a Europa": local poster Portuguese | Portuguese |
| [10.4126/FRL01-006433004](https://doi.org/10.4126/FRL01-006433004) | Local Poster | Registr dobrovolníků pro klinické studie vakcín proti COVID-19: "VACCELERATE - jednotný vstupní bod pro Evropu": local poster Czech | Czech |
| [10.4126/FRL01-006433008](https://doi.org/10.4126/FRL01-006433008) | Local Poster | Registre des volontaires pour les essais du vaccin COVID-19: "VACCELERATE - un point d' entrée unique pour l' Europe": local poster French | French |
| [10.4126/FRL01-006433020](https://doi.org/10.4126/FRL01-006433020) | Local Poster | Registro de voluntarios para ensayos de vacunas contra COVID-19: VACCELERATE - un Único Punto de Entrada para Europa": local poster Spanish | Spanish |
| [10.4126/FRL01-006433016](https://doi.org/10.4126/FRL01-006433016) | Local Poster | Registro dei volontari per la sperimentazione dei vaccini anti-Covid-19: "VACCELERATE - un punto d'accesso unico per l'Europa": local poster Italian | Italian |
| [10.4126/FRL01-006433006](https://doi.org/10.4126/FRL01-006433006) | Local Poster | Volunteer Registry for COVID-19 Vaccine Trials: "VACCELERATE - a Single Entry Point For Europe": local poster English | English |
| [10.4126/FRL01-006433002](https://doi.org/10.4126/FRL01-006433002) | Local Poster | Μητρώο εγγραφής εθελοντών για κλινικές δοκιμές εμβολίων COVID-19: "VACCELERATE - Ένα κοινό σημείο αναφοράς για την Ευρώπη": local poster Cypriot Greek | Greek |
| [10.4126/FRL01-006433012](https://doi.org/10.4126/FRL01-006433012) | Local Poster | Μητρώο εγγραφής εθελοντών για κλινικές δοκιμές εμβολίων COVID-19: "VACCELERATE - Ένα κοινό σημείο αναφοράς για την Ευρώπη": local poster Greek | Greek |
| [10.4126/FRL01-006433014](https://doi.org/10.4126/FRL01-006433014) | Local Poster | מרשם המתנדבים לניסויי חיסונים נגד COVID-19: local poster Hebrew: "VACCELERATE -נקודת כניסה אחת לאירופה" | Hebrew |
| [10.4126/FRL01-006432816](https://doi.org/10.4126/FRL01-006432816) | Poster | VACCELERATE Volunteer Registry: #bethemissingpiece - together we can tackle the COVID-19 pandemic - poster 1 | English |
| [10.4126/FRL01-006432943](https://doi.org/10.4126/FRL01-006432943) | Poster | VACCELERATE Volunteer Registry: #bethemissingpiece - together we can tackle the COVID-19 pandemic - poster 2 | English |
| [10.4126/FRL01-006432944](https://doi.org/10.4126/FRL01-006432944) | Poster | VACCELERATE Volunteer Registry: #bethemissingpiece - together we can tackle the COVID-19 pandemic - poster 3 | English |
| [10.4126/FRL01-006433037](https://doi.org/10.4126/FRL01-006433037) | Promotional Video | VACCELERATE project video: for adults | English |
| [10.4126/FRL01-006433038](https://doi.org/10.4126/FRL01-006433038) | Promotional Video | VACCELERATE promotional video: full version | English |
| [10.4126/FRL01-006433039](https://doi.org/10.4126/FRL01-006433039) | Promotional Video | VACCELERATE promotional video: work package leaders | English |
| [10.4126/FRL01-006433032](https://doi.org/10.4126/FRL01-006433032) | Puzzle | Kids Puzzle 1 - 3 (12 pieces) | English |
| [10.4126/FRL01-006433034](https://doi.org/10.4126/FRL01-006433034) | Puzzle | VACCELERATE VOLUNTEER REGISTRY Puzzle (25 pieces) | English |
| [10.4126/FRL01-006432953](https://doi.org/10.4126/FRL01-006432953) | Toolkit | VACCELERATE - the project toolkit | English |
| [10.4126/FRL01-006433036](https://doi.org/10.4126/FRL01-006433036) | Video | Meet the national coordinator for each participating country | English |
| [10.4126/FRL01-006433040](https://doi.org/10.4126/FRL01-006433040) | Video for Children | VACCELERATE video for children: English version | English |
| [10.4126/FRL01-006433041](https://doi.org/10.4126/FRL01-006433041) | Video for Children | VACCELERATE video for children: German version | German |
| [10.4126/FRL01-006433042](https://doi.org/10.4126/FRL01-006433042) | Video for Children | VACCELERATE video for children: Greek version | Greek |
| [10.4126/FRL01-006433043](https://doi.org/10.4126/FRL01-006433043) | Video for Children | VACCELERATE video for children: Spanish version | Spanish |
| [10.4126/FRL01-006433044](https://doi.org/10.4126/FRL01-006433044) | Video for Children | VACCELERATE video for children: Spanish version - boy & girls voices | Spanish |
| [10.4126/FRL01-006434909](https://doi.org/10.4126/FRL01-006434909) | Video for Children | VACCELERATE video for children: Italian version | Italian |
